# Cobalt nanoparticles induce lung injury, DNA damage and mutations in mice

**DOI:** 10.1186/s12989-017-0219-z

**Published:** 2017-09-18

**Authors:** Rong Wan, Yiqun Mo, Zhenyu Zhang, Mizu Jiang, Shichuan Tang, Qunwei Zhang

**Affiliations:** 1 Department of Pathology, Fujian Medical University, Fuzhou, People’s Republic of China; 2 Department of Environmental and Occupational Health Sciences, School of Public Health and Information Sciences, University of Louisville, 485 E. Gray Street, Louisville, KY 40202 USA; 3 Seven-year Program of Clinical Medicine, Fujian Medical University, Fuzhou, People’s Republic of China; 4 Department of Gastroenterology, Children’s Hospital, Zhejiang University, Hangzhou, People’s Republic of China; 5 Beijing Municipal Institute of Labor Protection, Beijing, People’s Republic of China; 6 Department of Preventive Medicine, Fujian Provincial Key Laboratory of Environment Factors and Cancer, School of Public Health, Fujian Medical University, Fuzhou, 350122 People’s Republic of China

**Keywords:** Cobalt nanoparticles, DNA damage, Histone H2AX phosphorylation, 8-hydroxy-2′-deoxyguanosine, Mutation, Oxidative stress, Titanium dioxide nanoparticles

## Abstract

**Background:**

We and other groups have demonstrated that exposure to cobalt nanoparticles (Nano-Co) caused oxidative stress and inflammation, which have been shown to be strongly associated with genotoxic and carcinogenic effects. However, few studies have reported Nano-Co-induced genotoxic effects in vivo. Here, we propose that Nano-Co may have high genotoxic effects due to their small size and high surface area, which have high capacity for causing oxidative stress and inflammation.

**Methods:**

*gpt* delta transgenic mice were used as our in vivo study model. They were intratracheally instilled with 50 μg per mouse of Nano-Co. At day 1, 3, 7 and 28 after exposure, bronchoalveolar lavage (BAL) was performed and the number of neutrophils, CXCL1/KC level, LDH activity and concentration of total protein in the BAL fluid (BALF) were determined. Mouse lung tissues were collected for H&E staining, and Ki-67, PCNA and γ-H2AX immunohistochemical staining. 8-OHdG level in the genomic DNA of mouse lungs was determined by an OxiSelect™ Oxidative DNA Damage ELISA Kit, and mutant frequency and mutation spectrum in the *gpt* gene were also determined in mouse lungs at four months after Nano-Co exposure by 6-TG selection, colony PCR, and DNA sequencing.

**Results:**

Exposure of mice to Nano-Co (50 μg per mouse) resulted in extensive acute lung inflammation and lung injury which were reflected by increased number of neutrophils, CXCL1/KC level, LDH activity and concentration of total protein in the BALF, and infiltration of large amount of neutrophils and macrophages in the alveolar space and interstitial tissues. Increased immunostaining of cell proliferation markers, Ki-67 and PCNA, and the DNA damage marker, γ-H2AX, was also observed in bronchiolar epithelial cells and hyperplastic type II pneumocytes in mouse lungs at day 7 after Nano-Co exposure. At four months after exposure, extensive interstitial fibrosis and proliferation of interstitial cells with inflammatory cells infiltrating the alveolar septa were observed. Moreover, Nano-Co caused increased level of 8-OHdG in genomic DNA of mouse lung tissues. Nano-Co also induced a much higher mutant frequency as compared to controls, and the most common mutation was G:C to T:A transversion, which may be explained by Nano-Co-induced increased formation of 8-OHdG.

**Conclusion:**

Our study demonstrated that exposure to Nano-Co caused oxidative stress, lung inflammation and injury, and cell proliferation, which further resulted in DNA damage and DNA mutation**.** These findings have important implications for understanding the potential health effects of nanoparticle exposure.

## Background

The application of nanotechnological products in human activities has been rapidly increasing in the past decade largely due to the unique properties of engineered nanoparticles. Of utmost interest are metal nanoparticles and their biological effects, since these nanoparticles have been widely used in cosmetics, medicine, electronics and industry. Cobalt is a transition and magnetic metal; cobalt nanoparticles (Nano-Co) have special characteristics, such as high surface area due to their small size, high magnetism and unique catalytic properties, etc. They are widely used in industrial applications such as magnetic tape, chemical catalysis, gas sensing equipment, coating, and light absorbance, as well as in medical biotechnology such as magnetic resonance imaging [1–3], and an experimental cancer treatment called magnetic hyperthermia, which uses the heat that nanoparticles produced when they are placed in an alternative magnetic field to kill cancer cells [2, 4, 5]. Thus, occupational and non-occupational exposure to metal nanoparticles is growing, and their potential health effects cannot be ignored. In fact, we and other in vitro studies have shown that exposure to cobalt or cobalt-containing nanoparticles caused oxidative stress, inflammation, and DNA damage [6–11]. In addition, some metal nanoparticles may have genotoxic and carcinogenic effects due to the chemical nature of the metal. Certain metal particles can generate reactive oxygen species (ROS) that can cause oxidative stress and DNA damage. For example, a couple of studies have shown that exposure to magnetite or carbon nanoparticles induced DNA mutations in the mouse lungs [12, 13]. Although some studies have shown the potential genotoxic effects of some nanomaterials, their mechanisms are still unclear. In this study, we selected Nano-Co as a ‘model’ metal nanoparticle because of its industrial interest and wide biological and medical applications. We examined its potential genotoxic effects and the possible mechanisms involved. Investigating the genotoxic effects and measuring the different types of DNA damage, such as gene mutation and DNA strand break formation, are important parts of evaluation and assessment of potential carcinogens.

It is well known that one initiating pathogenic effect of oxidative stress is inflammation, which can cause DNA damage and genotoxicity [14–18]. The association between genotoxicity and cancer is well known. For example, the carcinogenic effects of ionizing radiation, UV radiation and many chemical carcinogens are based on their ability to cause DNA damage and consequent gene mutation [15, 18, 19]. There are several excellent reviews regarding metals, oxidative stress and cancer [15–17]. It is generally accepted that excessive generation of ROS overwhelms the antioxidant defense system, causing oxidation of cellular biomolecules [18]. Free radicals cause oxidative modifications in DNA, including strand breaks and base oxidation. Among oxidative DNA damage products, 8-hydroxy-2′-deoxyguanosine (8-OHdG) is probably the most studied, due to its relative ease of measurement and pre-mutagenic potential [15, 16, 18, 20, 21]. Elevated 8-OHdG has been noted in numerous tumors, strongly implicating such damage in the etiology of cancer [15, 21]. Although uptake of nanoparticles by cells depends on material, size, shape, surface charge, and so on [22], previous studies have shown that metal nanoparticles may be able to penetrate into cells through passive diffusion, receptor-mediated endocytosis, and clathrin- or caveolae-mediated endocytosis, and subsequently enter the nucleus through diffusion across the nuclear membrane, or via the nuclear pore complexes. They may also become enclosed in the nucleus by a change following mitosis as the nuclear membrane dissolves during cell division and then reforms in each daughter [23–25]. If nanoparticles enter the nucleus, then direct interaction between nanoparticles and DNA or DNA-related proteins could lead to physical damage to the genetic material [24, 26].

Our and other previous in vitro studies have shown that exposure to cobalt nanoparticles or cobalt alloys such as cobalt-chromium alloy nanoparticles, caused DNA damage and cell transformation, and that ROS generation and oxidative stress were involved in those effects [10, 11, 27–30]. However, previous studies were conducted in cultured cells, and confirmation from animal experiments which are more relevant to human exposure is required. Therefore, the aim of present study is to explore the genotoxic effects of Nano-Co in vivo by using guanine phosphoribosyltransferase (*gpt)* delta transgenic mice which carry about 80 copies of the transgene, lambda EG10 DNA, on each chromosome 17 [31, 32]. When rescued phages are used to infect *E. coli* expressing Cre recombinase, they are converted into plasmids harboring the chloramphenicol (Cm) resistance and *gpt* genes. *Gpt* mutants are selected using plates containing Cm and 6-thioguanine (6-TG). And mutant frequency and mutant sequence analysis of *gpt* gene in the lung tissues from mice exposed to Nano-Co were evaluated. In addition, γ-H_2_AX immunostaining and 8-OHdG level in the genomic DNA in the lung tissues from mice exposed to Nano-Co were also determined.

## Methods

### Metal nanoparticles

Nano-Co and Nano-TiO_2_ with a mean diameter of 20 nm and 28 nm were provided by INABTA and Co., Ltd., Vacuum Metallurgical Co., Ltd., Japan. The characterization of these nanoparticles was described in our previous studies [11, 33]. Briefly, the diameter by transmission electron microscopy (TEM) is 10–40 nm for Nano-Co and 10–60 nm for Nano-TiO_2_, and the specific surface area is 47.9 m^2^/g for Nano-Co and 45.0 m^2^/g for Nano-TiO_2_. Nano-Co is composed of 85–90% metal cobalt and 10–15% Co_3_O_4_; Nano-TiO_2_ is composed of 90% anatase and 10% rutile. The size of particles and agglomerates in physiological saline was 260 nm for Nano-Co and 280 nm for Nano-TiO_2_, measured by dynamic light scattering (DLS). Nano-Co and Nano-TiO_2_ were dispersed in physiological saline and ultrasonicated for 10 min prior to each experiment. The solubility of Nano-Co and Nano-TiO_2_ in 1 × PBS and artificial alveolar fluid [34] was measured as previously reported [11, 35]. In brief, five 30 mg samples of Nano-Co or Nano-TiO_2_ were suspended in 30 ml of 1 × PBS or artificial alveolar fluid, respectively. After shaking for 48 h in a water bath at 37 °C, the samples were ultrasonicated for 30 min and then centrifuged at 12,000 g for 20 min. The supernatants were collected to determine the concentration of cobalt or titanium ion by inductively coupled plasma-atomic emission spectrometry (ICP-AES). The results were shown in Table 1.

**Table 1:** Ions released from metal nanoparticles in PBS and artificial alveolar fluid

Metal	PBS (ppm)	Artificial alveolar fluid (ppm)
Nano-Co	10.81 ± 0.62	16.85 ± 1.68
Nano-TiO_2_	<1.00	<1.00

### Animals

The *gpt* delta transgenic mice were originally obtained from Dr. Takehiko Nohmi at the National Institute of Health Science in Japan, which were established as described [30, 31, 33]. These mice carry about 80 copies of transgene, lambda EG10 DNA, on chromosome 17 in a C57BL/6 J background. The lambda EG10 DNA carries the *gpt* gene of *E. coli*. The enzyme encoded by *gpt* gene, guanine phosphoribosyltransferase, catalyzes phosphoribosylation of guanine, which is the obligatory step for the incorporation of guanine to DNA. This enzyme also phosphoribosylates 6-thioguanine (6-TG), which is toxic to cells when it is incorporated into DNA, thus allowing the selection of *gpt* mutants by 6-TG. In addition, the coding region of the *gpt* gene is 456 bp, which is convenient for the identification of mutation by DNA sequencing. The mice were bred and housed in an air-conditioned room (temperature of 20 ± 2 °C, relative humidity of 60 ± 10%) with a 12-h light and 12-h dark cycle environment, and with free access to food and water. Animal use was reviewed and approved by the University of Louisville Institutional Animal Care and Use Committee.

### Exposure of mice to metal nanoparticles

Female or male mice, 8–12 weeks old, weighted ~20-22 g for female and ~25-28 g for male, were exposed to nanoparticles by intratracheal instillation as described previously [33, 36, 37]. The intratracheal instillation model was selected instead of an inhalation study because the former is an easy and reliable method to identify nanoparticle toxicity and compare responses to different particle types [38]. Mice were grouped randomly (*n* = 4 ~ 8), but the ratio of female to male mice in each group was kept to ~1:1. Mouse neck skin was opened by a small midline incision and the trachea was isolated. 50 μL of physiological saline contained 50 μg of Nano-Co or Nano-TiO_2_ was instilled intratracheally by a syringe with a 28G1/2 needle, followed by 100 μL of air to ensure deposition into the lower airways. The skin was then closed with a monofilament nylon suture. Control mice were injected with physiological saline alone. At day 1 after exposure, mice lost 6.4 ± 1.8% of body weight for Nano-Co-treated group, but only 0.5 ± 1.3% for Nano-TiO_2_-treated group and no body weight loss was observed for control group. At 3 ~ 4 days after Nano-Co exposure, the mouse body weight started to bounce back. Only 1.0 ± 1.0% of body weight loss was observed at day 7 after Nano-Co exposure, and the body weight was slowly recovering until the end of the experimental period. The mice in the control and Nano-TiO_2_-treated groups grew normally. No mice died during the experimental period. The mice were sacrificed at day 1, 3, 7 and 28, or four months after instillation of metal nanoparticles.

### Preparation of bronchoalveolar lavage fluid (BALF)

The mice were sacrificed by overdose injection of tribromoethanol into the abdominal cavity and severing of abdominal aorta. The trachea was clearly visualized after skin and soft tissue dissection, and a 20 gauge cannula was inserted into the trachea. 0.8 mL of ice-cold 1xPBS containing 0.4 mM EDTA was used to lavage the lungs bilaterally. The lavage fluid was retrieved by gentle massage. The procedure was repeated another four times. The initially collected lavage fluid sample was centrifuged at 1600 rpm for 5 mins at 4°C. The supernatant was collected and stored at −80°C for subsequent analysis of biochemical markers. The cells in the BALF were pooled. The number of total cells was counted under a microscope using a hemacytometer. The cell differential count was evaluated on a cytospin slide stained with Giemsa May-Grünwald stains (Sigma-Aldrich, St. Louis, MO). A minimum of 400 cells were examined under a light microscope.

### Biochemical evaluation of BALF

The lactate dehydrogenase (LDH) activity in the BALF was measured by a LDH Cytotoxicity Detection Kit (TaKaRa Bio, Inc., Japan). The concentration of total protein in the BALF was determined by the Bradford method using Bio-Rad Protein Assay (Bio-Rad, Hercules, CA). The level of chemokine (C-X-C motif) ligand 1 (CXCL1)/keratinocyte chemoattractant (KC) was assessed by a Mouse CXCL1/KC PicoKine™ ELISA Kit (Boster Biological Technology, Pleasanton, CA).

### Histological examination

Mouse lung tissues were fixed in 10% neutral buffered formalin, dehydrated stepwise through ascending series of alcohol solutions and, finally, degreased in xylene. The tissues were then embedded in paraffin, sectioned at 5 μm by a microtome and stained with hematoxylin and eosin (HE) stains (Fisher, Fair Lawn, NJ).

### Immunohistochemistry

The expressions of Ki-67, PCNA and γ-H2AX were evaluated by immunohistochemistry in paraffin-embedded lung sections by a VECTASTAIN Elite ABC Kit (Vector Laboratories, Burlingame, CA). In brief, lung tissues were deparaffinized, hydrated, and immersed in 0.3% H_2_O_2_ in methanol for 30 min to inactivate endogenous peroxidase. For antigen retrieval, tissues were incubated in 10 mM sodium citrate (pH 6.0) with 0.05% Tween-20 at 95 °C for 30 min. Non-specific binding of antibodies was blocked by incubating tissues with Blocking Serum for 30 min at room temperature. Tissues were then incubated with anti-Ki-67 (Thermo Fisher, Waltham, MA), anti-PCNA (Santa Cruz, Dallas, TX), or anti-γ-H2AX (Millipore, Billerica, MA) antibody overnight at 4 °C. After being washed, tissues were subsequently incubated with biotinylated secondary antibody for 1 h, streptavidin-conjugated horseradish peroxidase for another 1 h, and 3, 3-diaminobenzidine (DAB) until desired stain intensity develops, with the washing between each step. Sections were counterstained with hematoxylin, mounted and examined under a light microscope. For quantitation of Ki-67, PCNA and γ-H2AX staining in mouse lung sections, numbers of both immunostaining positive and negative cells on three randomly selected high-power fields were counted and combined for each mouse. At least 600 cells per mouse were examined, and the percentage of immunostaining positive cells was calculated for each mouse (% positive cells = number of positive cells ⁄ number of total cells × 100) [39].

### Trichrome staining

Masson’s Trichrome for Connective Tissue kit (Electron Microscopy Sciences, Hatfield, PA) was used according to the manufacturer’s instructions with minor modifications. Lung sections were deparaffinized, hydrated to distilled water, and mordanted in Bouin’s Fixative at room temperature overnight to intensify the final coloration. After being washed, tissues were stained in Weigert’s Iron Hematoxylin Working solution for 4 min. After the wash, tissues were stained in Biebrich Scarlet-Acid Fuchsin for 4 min. After rinsing with deionized water, tissues were treated with Phosphomolybdic Acid/Phosphotungstic Acid for 15 min, followed by staining in Aniline Blue Solution for 15 min. After briefly being differentiated in 1% Acetic Acid, tissues were dehydrated through alcohol, cleared in xylene, and mounted.

### Determination of 8-OHdG level

8-OHdG level was determined in the genomic DNA of mouse lung tissues. Genomic DNA was purified by using a QIAamp DNA Mini kit (QIAGEN, Germantown, MD) according to the manufacturer’s instructions with minor modification. Briefly, 10–25 mg of lung tissues were cut into small pieces and lysed in 180 μL of Buffer ATL and 20 μL of proteinase K at 56°C water bath until the tissue was completely lysed. 4 μL of 100 mg/mL RNase A was added to degrade the RNA. 200 μL of Buffer AL was added to the solution and the tube was incubated at 70°C for 10 min. After being mixed with 200 μL of 100% ethanol, the solution was applied to a QIAamp Mini spin column. After being washed, the genomic DNA was dissolved in 200 μL of nuclease-free distilled water and its concentration was determined by a spectrophotometer. 8-OHdG level in genomic DNA was determined by OxiSelect™ Oxidative DNA Damage ELISA Kit (Cell Biolabs, San Diego, CA) according to the manufacturer’s instructions, and expressed as the amount of 8-OHdG (pg) in one microgram (μg) of genomic DNA.

### Determination of *gpt* mutant frequency

Genomic DNA was extracted from mouse lung tissues using a RecoverEase DNA Isolation Kit (Stratagene, La Jolla, CA), and Lambda EG10 phages were rescued using Transpack Packaging Extract (Stratagene) according to the manufacturer’s instruction. The *gpt* mutations were detected as described previously [12, 31, 40–42]. Briefly, *E.coli* YG6020 expressing Cre recombinase (provided by Dr. Nohmi) was infected with the rescued phages and spread on M9 salt plates containing chloramphenicol (Cm) and 6-thioguanine (6-TG). The plate was incubated at 37°C for 72 h, which enables selection of colonies harboring a plasmid carrying genes for chloramphenicol acetyltransferase (CAT) and a mutated *gpt*. Those (Cm + 6-TG)-resistant colonies, which contain a mutated *gpt*, were counted. The 6-TG-resistant phenotype of the colony was again confirmed by streaking *E.coli* cells on the (Cm + 6-TG) agar plate and the plate was incubated at 37°C for 72 h. To obtain the total number of Cm-resistant colonies, *E.coli* YG6020 was infected with an aliquot of rescued phage suspension and spread on M9 salt plates containing chloramphenicol (Cm) only, but without 6-TG. The plate was also incubated at 37°C for 72 h. Mutant frequency was calculated by dividing the number of colonies growing on (Cm + 6-TG) agar plate by the number on Cm agar plate as reported previously [12, 31, 40–42].

### Colony PCR and DNA sequencing analysis of *gpt* mutants

Colony PCR was performed to amplify a 739 bp DNA fragment containing the mutated *gpt* gene in the (Cm + 6-TG)-resistant colonies as described previously [41, 43]. Briefly, colonies on the (Cm + 6-TG) agar plates were picked and transferred to PCR tubes containing 25 μL of sterile water. After being incubated at 99°C for 5 min to lyse the cells and denature DNases, the tube was centrifuged to remove cell debris, and 5 μL of the solution was transferred to a new PCR tube for PCR amplification. PCR was performed on a Mastercycler (Eppendorf, Hauppauge, NY) for 35 cycles, each cycle using sequentially 94°C for 30 s, 58°C for 30 s, and 72°C for 1 min. The forward primer was: 5′-TACCACTTTATCCCGCGTCAGG-3′. The reverse primer was: 5′-ACAGGGTTTCGCTCAGGTTTGC-3′. The amplified PCR products were checked by agarose gel electrophoresis, and sent to DNA core facility at University of Louisville for sequencing. The sequencing primers were either 5′-GAGGCAGTGCGTAAAAAGAC-3′ or 5′-CTATTGTAACCCGCCTGAAG-3′ as described previously [41].

### Statistical analysis

Data were expressed as the mean ± SE. Differences among groups were evaluated by one-way analysis of variance (ANOVA) followed by Dunnett’s t-test or Bonferroni t-test. If necessary, transformation (logarithmic or square root) of data was used to achieve normally distributed data. If a *p* value was less than 0.05, a difference was considered statistically significant. Statistical analyses were carried out using SigmaPlot 13.0 software (Systat Software, Inc., San Jose, CA).

## Results

### Cellular and biochemical constituents in BALF

**Fig. 1:**
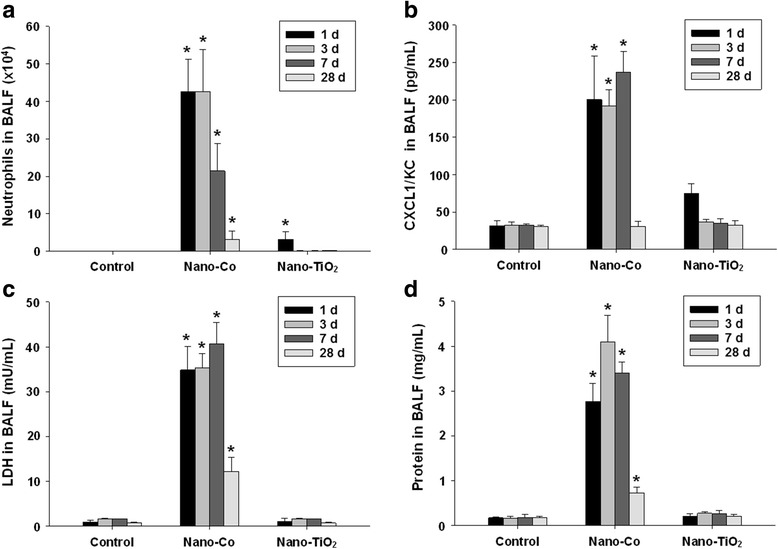
Cellular and biochemical constituents in bronchoalveolar lavage fluid (BALF). Mice were intratracheally instilled with 50 μg per mouse of Nano-Co or Nano-TiO_2_. Control mice were instilled with physiological saline. At day 1, 3, 7 and 28 after exposure, BALF was collected and the number of neutrophils (**a**), CXCL1/KC level (**b**), LDH activity (**c**), and the concentration of total protein (**d**) in BALF were determined. Values are the mean ± SE of 4 ~ 8 mice. * Significant difference from the control, *p* < 0.05

To investigate whether exposure to metal nanoparticles caused lung inflammation and lung injury, the cellular and biochemical constituents in BALF obtained from mice exposed to metal nanoparticles at different doses and time points were evaluated. Our results showed that intratracheal instillation of 50 μg per mouse of Nano-Co caused severe acute inflammatory response and lung injury, which was reflected by a marked increase in the total number of neutrophils, CXCL1/KC level, LDH activity and concentration of total protein in BALF obtained from mice at as early as day 1 after exposure (Fig. 1a–d). These parameters were maintained at a higher level at day 3 and day 7 after Nano-Co exposure, but declined and returned toward control levels by day 28 (Fig. 1a–d). There was a dose-related increase in these parameters when mice were exposed to 50 and 100 μg per mouse of Nano-Co (Data not shown). However, exposure to Nano-TiO_2_ only caused a slight increase in the number of neutrophils and CXCL1/KC level at day 1 after exposure, but both returned to control levels at day 3 after exposure and beyond (Fig. 1a–b). Nano-TiO_2_ exposure did not have any effects on the LDH activity and the concentration of total protein in the BALF (Fig. 1c–d).

### Histopathology

**Fig. 2:**
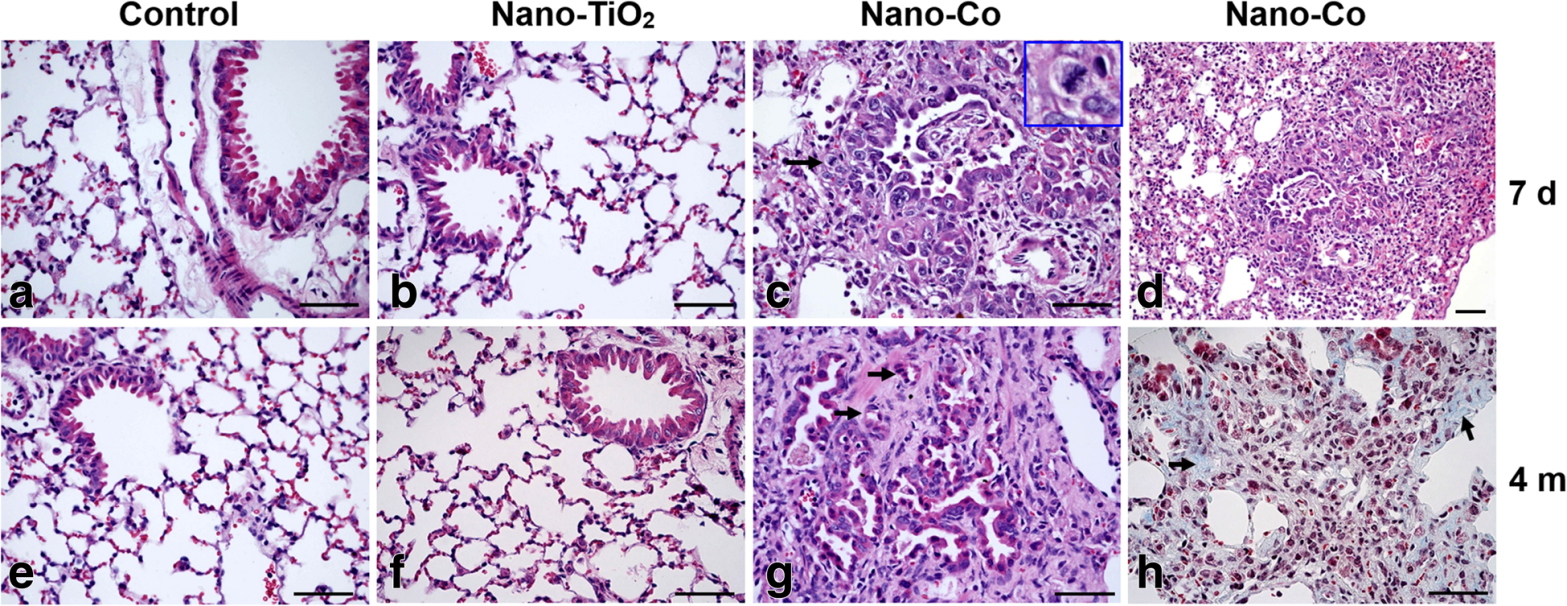
Histological changes in lungs of mice 7 days and 4 months after metal nanoparticle exposure. Mice were instilled intratracheally with 50 μg per mouse of Nano-Co or Nano-TiO_2_. Control mice were instilled with physiological saline. Lung sections collected from mice 7 days (**a**–**d**) and 4 months (**e**–**h**) after exposure were analyzed by H&E staining (**a**–**g**) or trichrome staining (**h**). **a** & **e** show normal lung parenchymal structure in control mice. (**c** & **d**) show severe acute lung inflammation at day 7 after Nano-Co exposure, which was reflected by infiltration of a large amount of neutrophils and macrophages into the alveolar space and septa, focal alveolar epithelial cell hyperplasia, and thickening of the alveolar wall. Arrow in (**c**) shows a mitotic hyperplastic alveolar epithelial cell, which was enlarged at the top right corner of (**c**). **g** shows extensive interstitial fibrosis and bronchiolization of the alveoli (arrows in **g**) in the lungs of mice at 4 months after Nano-Co exposure. **h** shows increased collagen deposition (blue staining) in the alveolar septa by trichrome staining. However, Nano-TiO_2_ instillation did not cause these effects (**b** & **f**). Scale bar: 50 μm

In order to observe the histopathological changes in the lungs, H&E staining was performed on the lung sections collected from mice after metal nanoparticle exposure. In the control mice, which were instilled with physiological saline, normal lung parenchymal structures were observed (Fig. 2a & e). Instillation of Nano-Co induced severe acute lung inflammation at as early as day 1 after exposure, as evident by an increase in the influx of neutrophils (data not shown). At day 7 after exposure, infiltration of a large number of neutrophils and macrophages into the alveolar space and interstitial tissues, focal alveolar epithelial cell hyperplasia, and thickening of the alveolar septa were observed (Fig. 2c & d). At 4 months after instillation of Nano-Co, extensive interstitial fibrosis and infiltration of inflammatory cells into the alveolar space and interstitial tissues were observed in the mouse lungs (Fig. 2g). In the thickened alveolar septa, bronchiolization of the alveoli which was characterized by replacement of alveolar epithelial cells with bronchiolar-type epithelium (arrows in Fig. 2g), micro-papillomatosis, and emphysematous changes were also observed (Fig. 2g). The Nano-Co-induced lung interstitial fibrosis was further confirmed by Masson’s trichrome staining, which showed an increase in the collagen deposition and proliferation of interstitial cells in the lung tissues (Fig. 2h). In contrast, Nano-TiO_2_ instillation only caused none or a mild inflammatory response in the mouse lungs, and there was no obvious fibrotic response in the lungs at 4 months after Nano-TiO_2_ exposure (Fig. 2b & f).

### Immunohistochemical staining for cell proliferation markers

**Fig. 3:**
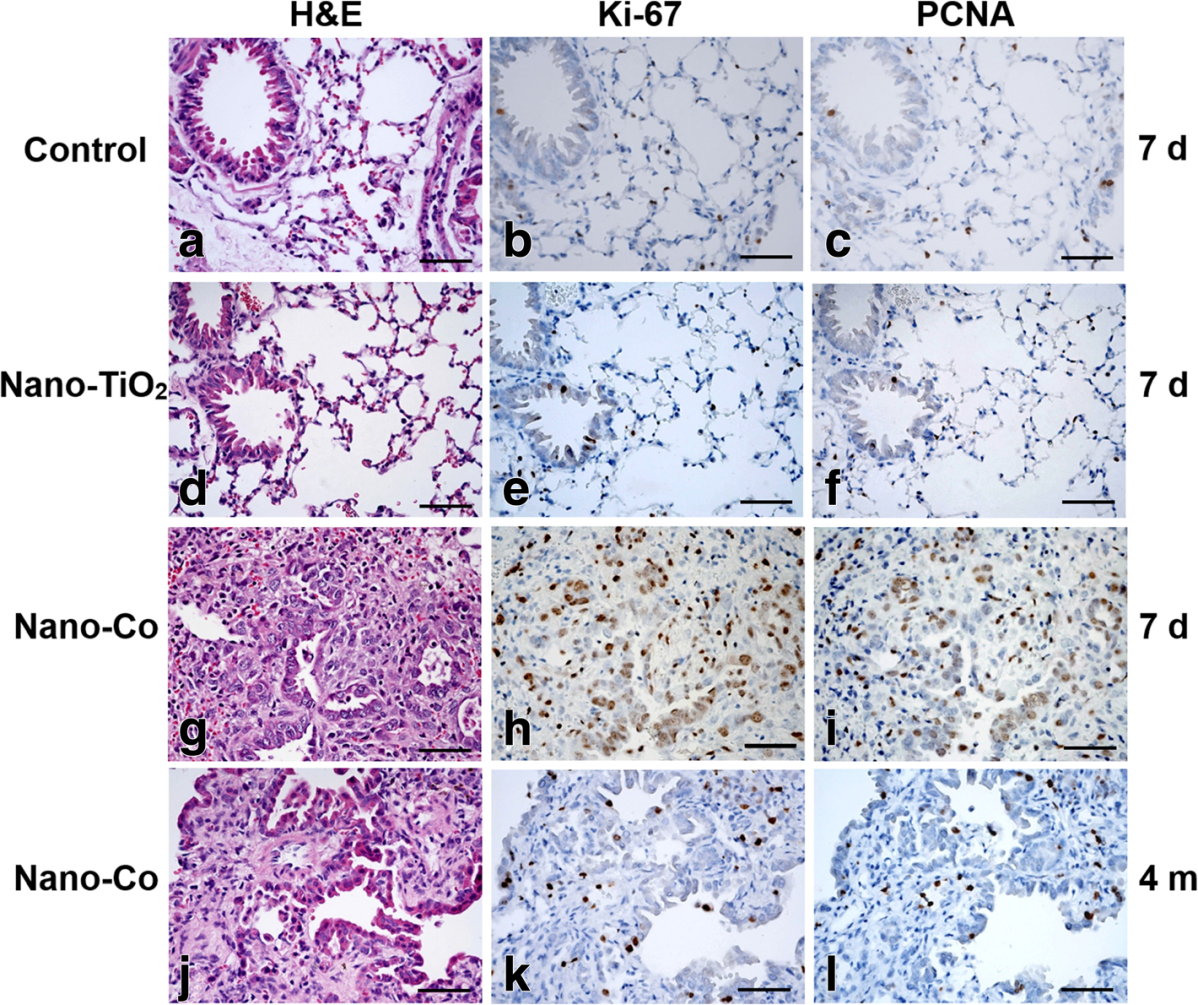
Increased Ki-67 and PCNA expression in mouse lungs after Nano-Co exposure. Mice were instilled intratracheally with 50 μg per mouse of Nano-Co or Nano-TiO_2_. Control mice were instilled with physiological saline. Lung sections collected from mice 7 days (**a**–**i**) and 4 months (**j**–**l**) after exposure were analyzed by immunohistochemistry staining. Scale bar: 50 μm

The expression of Ki-67 and PCNA, genes associated with cell proliferation, was determined in the lung sections by immunohistochemical staining. Our results showed that both Ki-67 and PCNA positive staining were located in the nucleus. The Ki-67-positive (33.8 ± 6.5%) or PCNA-positive (20.6 ± 4.5%) cells were significantly increased at day 7 after Nano-Co exposure, as compared to that in the control or Nano-TiO_2_-treated group (Fig. 3h & i). In the control or Nano-TiO_2_-treated group, only a few Ki-67-positive (Control: 7.9 ± 2.0%, Nano-TiO_2_: 8.7 ± 2.1%) (Fig. 3b & ess) or PCNA-positive (Control: 5.7 ± 1.6%, Nano-TiO_2_: 6.3 ± 1.8%) (Fig. 3c & f) cells were observed. By 4 months after instillation, Nano-Co still caused increase in the number of Ki-67-positive (11.7 ± 1.7%) or PCNA-positive (10.8 ± 2.3%) cells as compared to the control or Nano-TiO_2_-treated group, but much less than that at day 7 after Nano-Co exposure (Fig. 3k & l).

### Immunohistochemical staining for DNA damage marker

**Fig. 4:**
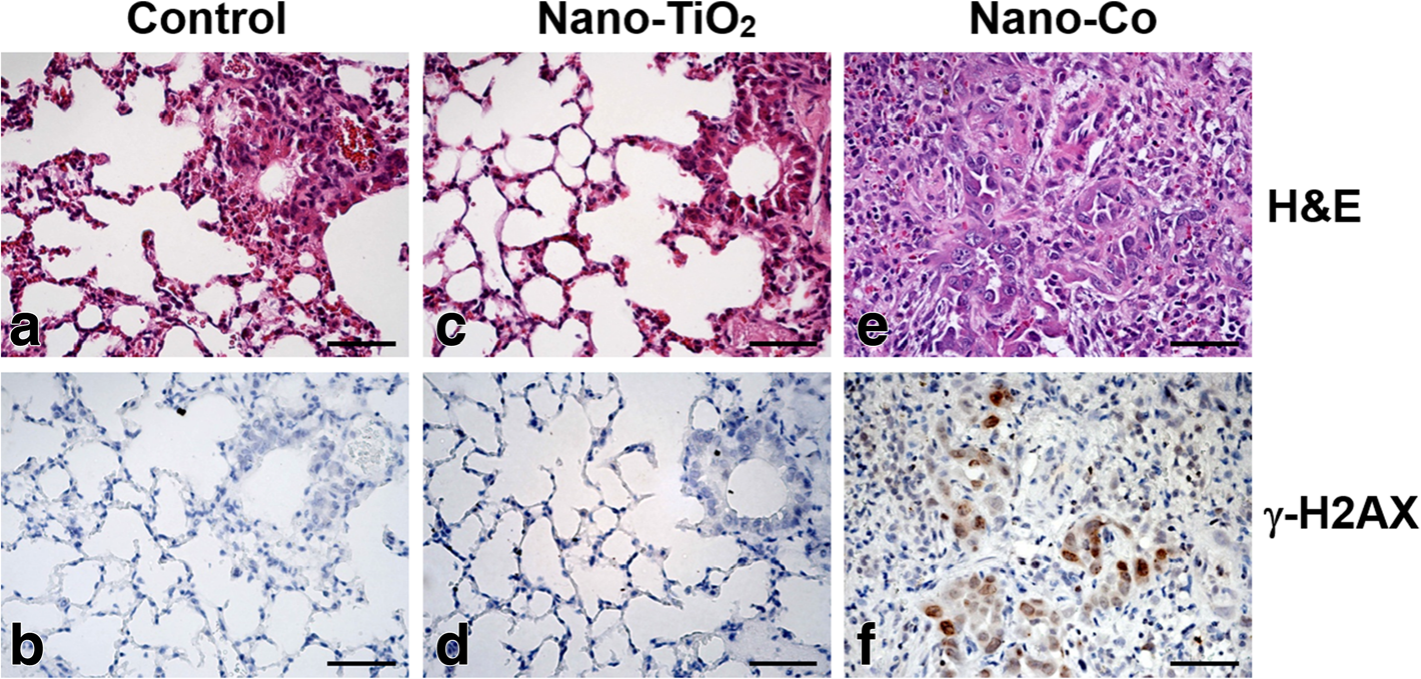
Increased γ-H2AX immunostaining in mouse lungs 7 days after Nano-Co exposure. Mice were instilled intratracheally with 50 μg per mouse of Nano-Co or Nano-TiO_2_. Control mice were instilled with physiological saline. Lung tissues collected from mice 7 days after exposure were analyzed by H&E staining (**a**, **c**, **e**) and γ-H2AX immunohistochemistry staining (**b**, **d**, **f**). Scale bar: 50 μm

H_2_AX phosphorylation at Ser139 correlates very closely with each double-strand DNA break (DSB), therefore, phospho-H_2_AX (γ-H_2_AX) is a sensitive marker for DNA damage and can be used to assess the effects and timing of DNA damage-inducing and DNA repair agents [44, 45]. In the present study, in order to examine the potential genotoxic effects of metal nanoparticles, γ-H_2_AX immunostaining was evaluated in lung tissues from mice exposed to metal nanoparticles. At day 7 after instillation, the lungs from Nano-Co-treated mice showed a significant increase in γ-H_2_AX-positive nuclei as compared to the physiological saline-instilled control group (15.3 ± 3.5% vs. 1.3 ± 0.3%, *p* < 0.01) (Fig. 4b & f). Nano-TiO_2_-treated mice demonstrated a slight but not significant increase in γ-H_2_AX-positive nuclei (1.9 ± 0.4%), less pronounced compared to the Nano-Co-treated group (Fig. 4d). At 4 months after instillation, Nano-Co still caused an increase in γ-H_2_AX-positive nuclei as compared with the control and Nano-TiO_2_-treated mice (Data not shown).

### 8-OHdG level in the lung tissues

**Fig. 5:**
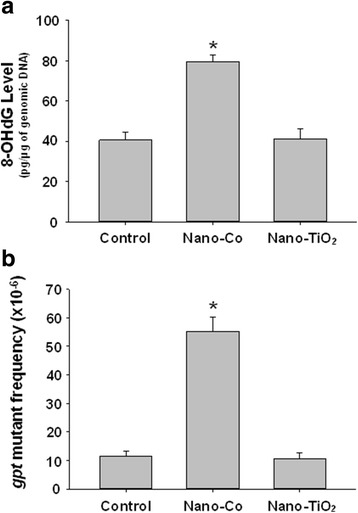
8-OHdG level (**a**) and *gpt* mutant frequency (**b**) in the genomic DNA of lungs from mice exposed to metal nanoparticles. Mice were instilled intratracheally with 50 μg per mouse of Nano-Co or Nano-TiO_2_. Control mice were instilled with physiological saline. Lung tissues were collected four months after exposure. Data are mean ± SE of 4 ~ 6 mice. * *p* < 0.05 vs. Control

The level of 8-OHdG, a biomarker of oxidative DNA damage caused by reactive oxygen species (ROS), in the lung tissues obtained from mice 4 months after Nano-Co and Nano-TiO_2_ treatment was determined by ELISA. Our results showed that the level of 8-OHdG was 79.4 ± 3.4 pg per μg of genomic DNA in the Nano-Co-treated group, which was significantly higher than that in the control group (40.6 ± 3.9 pg per μg of genomic DNA) and in the Nano-TiO_2_-treated group (41.3 ± 4.7 pg per μg of genomic DNA) (Fig. 5a).

### *gpt* mutant frequency after metal nanoparticle instillation

To determine the mutagenic effects of metal nanoparticles, *gpt* delta transgenic mice were exposed to Nano-Co or Nano-TiO_2_ at a dose of 50 μg by single intratracheal instillation. At 4 months after exposure, mice were sacrificed and *gpt* mutations in the lungs of mice were analyzed as described in the Materials and Methods section. Table 2 and Fig. 5b showed the mutant frequencies (MFs) of *gpt* genes in the lungs. The background MF of lungs was 11.6 ± 1.8 × 10^−6^. MFs in the lungs induced by Nano-Co were increased by 4.8-fold as compared with physiological saline-instilled animals. However, exposure to Nano-TiO_2_ did not cause significant increase in MFs in the lungs.

**Table 2:** Mutant frequency (MF) of *gpt* gene in the lungs of control and metal nanoparticle-exposed mice

Treatment	Sex	No. of rescued colony	No. of mutant	MF (×10^−6^)	Average of MF ± SE (x10^-6^)
Control	F	477,000	5	10.5	11.6 ± 1.8
	F	540,000	9	16.7	
	M	229,500	2	8.7	
	M	1,429,500	15	10.5	
Nano-Co	F	397,500	20	50.3	55.2 ± 5.0^*^
	F	684,750	27	39.4	
	F	408,000	22	53.9	
	M	415,500	20	48.1	
	M	236,250	17	72.0	
	M	311,000	21	67.5	
Nano-TiO_2_	F	775,500	13	16.8	10.7 ± 1.9
	F	798,000	10	12.5	
	M	1,117,500	7	6.3	
	M	423,000	3	7.1	
	M	451,500	5	11.1	

Mice were instilled intratracheally with 50 μg per mouse of Nano-Co or Nano-TiO_2_. Control mice were instilled with physiological saline. Lung tissues were collected four months after exposure. * *p* < 0.05 vs. Control

### Mutation spectrum induced by metal nanoparticles

To examine the mutational characteristics induced by metal nanoparticles, 6-TG resistant *gpt* mutants were sequenced and analyzed. 62 independent 6-TG resistant mutants derived from Nano-Co, 38 from Nano-TiO_2_, and 19 from physiological saline instilled mice were identified. Classes of mutations found in the *gpt* gene were listed in Table 3. Base substitutions predominated with both metal nanoparticle-induced and spontaneous cases. No A:T to T:A and G:C to C:G transversions were detected in physiological saline-instilled control group, indicating that these types of mutations were rare events in the spontaneous mutations. However, G:C to A:T transition appears a common event in the spontaneous mutations. Interestingly, G:C to T:A transversion significantly increased in Nano-Co-treated group as compared with the control or Nano-TiO_2_-treated group, suggesting Nano-Co exposure can cause G:C to T:A transversion. Other types of mutations, including A:T to G:C transition, A:T to C:G transversion, deletions and insertions, were not significantly observed in both nanoparticle-treated and control groups.

**Table 3:** Summary of *gpt* mutations in the lungs of control and metal nanoparticle-exposed mice

Type of mutation in *gpt*	Control		Nano-Co		Nano-TiO_2_	
	No.	%	No.	%	No.	%
Transition						
G:C to A:T	6	31.6	14	22.6	13	34.2
A:T to G:C	2	10.5	1	1.6	6	15.8
Transversion						
G:C to T:A	6	31.6	38	61.3	11	28.9
G:C to C:G	0	0	1	1.6	0	0
A:T to T:A	0	0	1	1.6	0	0
A:T to C:G	2	10.5	2	3.2	3	7.9
Deletions	1	5.3	1	1.6	1	2.6
Insertions	2	10.5	2	3.2	3	7.9
Others	0	0	2	3.2	0	0
Total	19	100	62	100	38	100

Mice were instilled intratracheally with 50 μg per mouse of Nano-Co or Nano-TiO_2_. Control mice were instilled with physiological saline. Lung tissues were collected four months after exposure

## Discussion

Cobalt compounds (metal, salts, oxides, and alloys) are widely used in various industrial, medical and military applications. Exposure to cobalt and its compound particles can cause pulmonary fibrosis, interstitial pneumonitis and asthma [46–50]. Cobalt compounds are listed as probable or possible human carcinogens by IARC [47]. Chronic inhalation exposure to cobalt metal and cobalt sulfate caused lung cancer in rats and mice, as well as systemic tumors in rats [47–50]. Cobalt nanoparticles (Nano-Co) have been widely used in the industries, for example, as an alloying element in super alloys and magnetic and hard-metal alloys, as pigments in the glass and ceramics industries, as catalysts in the oil and chemical industries, as paint and printing ink driers, and as trace metal additives for agricultural and medical uses [1, 2, 5]. Therefore, the use of Nano-Co has grown in the last decades in many areas, requiring the scientific community access their toxicological, genotoxic and carcinogenic potential. Nano-Co may have an unpredictable impact on human health, since traditional toxicological knowledge, based on data derived from materials in their bulk form, is not applicable in the nano-size scale. We and other previous studies have shown that exposure to Nano-Co caused lung inflammation in the rats, and Nano-Co induced more severe lung inflammation than standard-sized (5 μM) cobalt particles [33, 37]. In addition, exposure to Nano-Co caused DNA damage and genotoxic effects in hepatocarcinoma cells, lung adenocarcinoma cells (A549), fibroblasts, bronchial epithelial cells (BEAS-2B) [11, 28, 50–52]. However, previous studies on DNA damage and genotoxic effects of Nano-Co were limited to in vitro studies. Only one study was found to focus on the genotoxicity of cobalt ferrite nanoparticles in the mouse liver, in which they demonstrated that exposure to cobalt ferrite nanoparticles caused increased expression of critical genes related to oxidative stress, apoptosis, DNA damage and cell damage [53]. However, no in vivo study was found to focus on the genotoxicity of Nano-Co in lungs. Our present study investigated whether exposure to metal nanoparticles, such as Nano-Co or Nano-TiO_2_, resulted in lung inflammation, DNA damage and DNA mutation in the mice by using *gpt* delta transgenic mice.

Although exposure to some metal nanoparticles, such as TiO_2_, has been identified as an occupational workplace hazard by the National Institutes of Occupational Safety and Health [54], exposure limits for cobalt nanoparticles remain undefined. Daily occupational exposures likely vary, as it is extremely difficult to predict an accurate exposure given the variability in dust generation and ventilation among worksites. Therefore, it is difficult to estimate what a relevant cobalt nanoparticle dosage or concentration should be for experimental models. The existing exposure limits for cobalt metal and ionic cobalt are difficult to translate to cobalt nanoparticle exposure; ionic cobalt is soluble in physiological fluids and cell culture buffers, and exposure modality would experience different physiological effects than exposure to a solid (non-soluble) cobalt nanoparticles. Previous studies used 10 mM cobalt chloride (CoCl_2_) in 25 μL volume to treat mice by oropharyngeal aspiration for 5 days on, 2 days off, and another 5 days on. This cobalt concentration corresponds to 60 μg daily exposure per mouse [55, 56]. The doses of metal nanoparticles we used in our in vivo model (50 μg per mouse) is much lower than the doses used in a previous study with cobalt oxide nanoparticle (Nano-CoO) exposure [57]. In that study, the investigators reported that inhalation of Nano-CoO at 10 mg/m^3^ (low dose) or 30 mg/m^3^ (high dose) for a duration of 6 h/day for 4 consecutive days, induced lung injury. For a mouse of 25 g in size, assuming the respiratory rate is ~163 per minute, the tidal volume is ~0.15 ml, and the respiratory deposition is ∼40%, the mouse would inhaled about 352 μg (low dose) and 1056 μg (high dose) of Nano-CoO, and 141 μg (low dose) and 423 μg (high dose) of Nano-CoO would deposit in the lungs. If taking into account the mass of Co in the CoO, there were 111 μg (low dose) and 333 μg (high dose) of Co deposited. The particles inhaled into the lungs could accumulate over time. Accidental exposure to metal nanoparticles at the workplace also cannot be ignored. There were no reports found about accidental Nano-Co exposure, however, there was a report about accidental exposure to nickel nanoparticles [58]. In that report, a worker was accidentally exposed to ~1 g of nickel nanoparticles during his 90 min of operating the process. He died from acute respiratory distress syndrome. Therefore, the dose we used in this study was not unreasonable, although using lower doses of Nano-Co in our future studies will boost our findings.

Analysis of cellular damage and biochemical profile of BALF after exposure to pulmonary toxin is a useful method for determining the inflammatory response and injury of lungs [59, 60]: it can be used to assess lung inflammation and injury induced by various particles including nanoparticles [33, 36, 37]. For example, LDH is a cytoplasmic enzyme that released extracellularly by damaged cells. Therefore, the LDH activity in BALF reflects the degree of damage to cells and tissues [33, 61, 62]. Accumulation of neutrophils and macrophages in the lungs are key events in the inflammatory response to inhaled particles [60]. We have performed dose- and time-response studies to evaluate the pulmonary toxic effects of metal nanoparticles. Our results showed that Nano-Co caused severe acute lung inflammation which was reflected by increased number of neutrophils in BALF obtained from mice at day 1, 3, and 7 after exposure to 50 μg of Nano-Co. This was accompanied by cell damage, as shown by increased LDH activity and total protein concentration in the BALF. Exposure to Nano-Co also caused a significant increase in CXCL1/KC level in BALF. CXCL1/KC is a neutrophil chemokine that is known to play an important role in neutrophil recruitment in a variety of direct and indirect lung injury models including sepsis, pancreatitis, and ventilator-induced lung injury [63]. Therefore, the increased CXCL1/KC level in the BALF may exacerbate Nano-Co-induced pulmonary inflammatory response by recruiting and activating neutrophils in the lungs through movement of circulating leukocytes to the lungs. Nano-Co-induced lung inflammation was further confirmed by histopathological examination, which revealed an influx of a large amount of neutrophils and macrophages into the pulmonary parenchyma after Nano-Co treatment. Macrophages are believed to be among the first and primary cell types that process nanoparticles, mediating host inflammatory and immunological biological responses. Our current results and the results from previous intratracheal instillation and inhalation studies in rats [37, 64] demonstrated that exposure to Nano-Co induced severe and persistent pulmonary inflammation, even at a low mass doses. This kind of persistent inflammation is consistent with particles of high pathogenicity such as quartz [61] and Nano-Ni [33, 38]. Instillation of bolus doses of non-pathogenic particles, such as Nano-TiO_2_ caused only transient inflammation that resolved in a few days post-instillation [33].

Oxidative stress is considered to be an important mechanism for particle-induced health effects. Exposure to some types of nanoparticles induce oxidative stress in cells, and activation of oxidative stress-responsive transcription factors such as NF-ĸB and AP-1, together with the depletion of antioxidant defenses, can lead to the release of pro-inflammatory cytokines [24]. It is generally accepted that oxidative stress eventually causes DNA damage, which plays an important role in the development of carcinogenesis [15, 16, 21]. 8-OHdG is a representative oxidative product of guanine and a highly mutagenic lesion that causes mispairing of 8-OHdG with deoxyadenosine (dA). It is a good marker for oxidative stress induced DNA damage [21]. The major problem associated with the measurement of oxidative DNA damage is the possibility of artifactual formation of DNA oxidative products during the isolation of DNA and during the analysis of 8-OHdG. However, it is important to note that, even if an oxidation occurs during derivatization, the differences in the levels of 8-OHdG between the control and the Nano-Co-treated groups observed in our study should not be artifactual, since the samples were treated in an identical fashion. Our results showed that exposure of mice to Nano-Co, but not Nano-TiO_2_, caused increased levels of 8-OHdG in genomic DNA of lungs, suggesting that Nano-Co-induced oxidative stress may be involved in Nano-Co-induced DNA damage in vivo.

The repair mechanisms for a specific type of DNA damage, such as double-strand DNA breaks (DSBs), involve the phosphorylation of H2AX (γ-H2AX) [44]. γ-H2AX was originally identified as an early event after the direct formation of DSBs by ionizing radiation [65]. It is now also considered to occur after the indirect formation of DSBs caused by the collision of the replication forks at sites of DNA damage including DNA adducts, single strand breaks (SSBs) and crosslinking, and the repair of the damage [45]. It was reported that γ-H2AX can be generated following the exposure of cells to various environmental factors and agents such as cigarette smoke, polycyclic aromatic hydrocarbons (PAHs), benzene metabolites, etc. [66, 67]. Therefore, studies using γ-H2AX might help to clarify the genotoxicity of exposure to various metal nanoparticles. Previous studies showed that the genotoxic potential of various nanoparticles as determined by γ-H2AX quantification in in vitro experiments was different with exposure to alumina (Al_2_O_3_) ceramic or cobalt–chromium metal nanoparticles, carbon nanotubes, and silica [68, 69]. Our previous studies have shown that γ-H2AX is a sensitive marker for DNA damage induced by Nano-Co in vitro [11]. In this study, we found that γ-H2AX-positive nuclei were significantly increased in the lungs from mice exposed to even one single dose of Nano-Co, as compared to mice exposed to Nano-TiO_2_ or physiological saline control. Nano-Co-induced DNA damage may be due to Nano-Co-induced oxidative stress and persistent inflammation in the lungs. Nanoparticles are not as readily phagocytized by alveolar macrophages as larger particles and can penetrate much more rapidly through the epithelium to interstitial tissues, which may result in persistent or progressive lung inflammation. Continued inflammation has been reported in an animal exposure model using asbestos and silica, thus persistent inflammation is an important feature in the formation of irreversible chronic lesions [70, 71]. Our experiments revealed that exposure to Nano-Co caused persistently increased numbers of neutrophils and 8-OHdG in the lungs. Therefore, Nano-Co-induced DNA damage in vivo might occur through a secondary genotoxic mechanism associated with inflammation and involving oxidative stress.

One method to assess the proliferative activity of cells is the immunohistochemical detection of cell cycle-specific antigens. For example, Ki-67 and PCNA proteins are standard markers of proliferation that are commonly used to assess the growth fraction of a cell population. Previous studies showed that the presence of the Ki-67 antigen in the nuclei of cells in all phases of the cell cycle, whereas it was not expressed in quiescent or resting cells in the G0 phase [72]. This feature makes this protein an excellent marker for determining the growth fraction of a given cell population [73–75]. In addition, the expression of Ki-67 is affected by external factors such as nutrient deprivation, which could lead to underestimation of the number of cycling cells [76]. Another widely used marker, PCNA, is a less specific proliferation marker because of its redundant role in DNA repair. Thus, immunohistochemical detection of PCNA may not only detect actively dividing cells, but also those in the process of DNA repair [77]. PCNA plays important roles in the metabolism of nucleic acid. Its main function is in DNA replication, but it is also involved in DNA excision repair, cell cycle control, chromatin assembly, and RNA transcription [78]. Our present study has demonstrated that Nano-Co-treated animals exhibited a significantly elevated proliferation at day 7 after treatment. Although the proliferation decreased at 4 months after exposure, mouse lungs still exhibited a significantly increased number of Ki-67-positive or PCNA-positive cells.

**Fig. 6:**
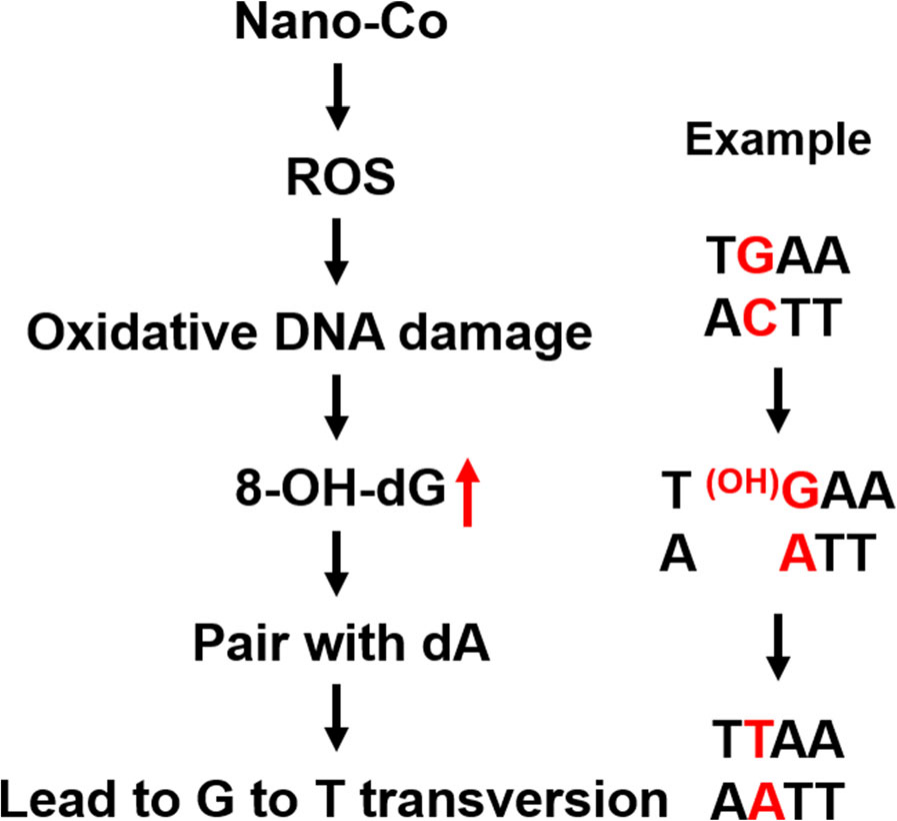
A schematic diagram of pathways that may be involved in Nano-Co-induced DNA mutation. Exposure to Nano-Co caused oxidative stress, which may further result in oxidative DNA damage and increased 8-OH-dG formation. 8-OHdG can pair with dA, thus leading to G:C to T:A transversion

Our results clearly showed that exposure to Nano-Co caused cell proliferation and DNA damage in the mice. Proliferation is an important prerequisite for manifestation of the genomic mutations. To determine whether exposure to metal nanoparticles caused mutagenic effects on the lungs, *gpt* gene mutations were determined and analyzed. We found that Nano-Co demonstrated mutagenicity in the *gpt* delta transgenic mouse system. The MFs of *gpt* gene were significantly increased in the lungs of *gpt* delta transgenic mice with exposure to Nano-Co, but not to Nano-TiO_2_ or to physiological saline. To explore the mechanisms involved in the Nano-Co-induced increase in MFs, we analyzed the mutation spectra using a PCR-direct sequencing method. The most prominent mutation type induced by Nano-Co was G:C to T:A transversion (38/62, 61.3%), which was only 31.6% (6/19) in physiological saline-treated control group and 28.9% (11/38) in Nano-TiO_2_-treated group. 8-OHdG contributes to G:C to T:A transversion upon DNA replication [79]. 8-OHdG is one of the multiple oxidative damages induced in DNA by OH· [80, 81]. This lesion represents 30–50% of the total base modification products induced by OH·-producing models and therefore is considered a ‘fingerprint’ of OH· attack on DNA [82]. Our previous in vitro and current in vivo studies showed that exposure to Nano-Co caused reactive oxygen species (ROS) generation and DNA damage, including the increased 8-OHdG level [11]. Transition metals including iron (Fe), copper (Cu), chromium (Cr), vanadium (V), and silica (Si) are involved in ROS generation via mechanisms such as Haber-Weiss and Fenton-type reactions [83, 84]. Since cobalt has similar chemical properties to iron, it is reasonable to propose Fenton like mechanisms for the production of ROS by cobalt. In fact, Nano-Co has been reported to induce oxidative stress (O_2_ ∙ − and OH∙) via Fenton-type reaction [85]. Nano-Co also caused accumulation of macrophages and leucocytes in the lungs which may also generate ROS [33, 37]. Fig. 6 is a schematic diagram showing the involvement of Nano-Co-induced oxidative stress in the Nano-Co-induced increased G:C to T:A transversion.

## Conclusions

Taken together, our study demonstrated that exposure to Nano-Co caused oxidative stress, lung inflammation and injury, and cell proliferation, which further resulted in DNA damage and DNA mutation. Our results also showed that Nano-Co induced a much higher mutant frequency as compared to controls, and the most common mutation was G:C to T:A transversion, which may be explained by Nano-Co-induced increased formation of 8-OHdG. These results represent the first comprehensive in vivo genotoxicity of Nano-Co. The results provide further understanding of the potential genotoxic effects of metal nanoparticle exposure. Further studies of the mechanism of genotoxicity and application routes other than intra tracheal instillation are needed. Finally, exposure levels of Nano-Co in the working environment should be determined.

## Abbreviations

6-TG: 6-thioguanine
8-OHdG: 8-hydroxy-2′-deoxyguanosine
BALF: Bronchoalveolar lavage fluid
CXCL1/KC: Chemokine (C-X-C motif) ligand 1/keratinocyte chemoattractant
DSBs: Double-strand breaks
gpt: Guanine phosphoribosyltransferase
LDH: Lactate dehydrogenase
MF: Mutant frequency
Nano-Co: Cobalt nanoparticles
Nano-TiO_2_: Titanium dioxide nanoparticles
PCNA: Proliferating cell nuclear antigen
ROS: Reactive oxygen species
